# Neuroimaging in an older adult inpatient psychiatric unit

**DOI:** 10.1192/bjo.2021.286

**Published:** 2021-06-18

**Authors:** Aoife Nechowska

**Affiliations:** Surrey and Borders Partnership NHS Foundation Trust

## Abstract

**Aims:**

This audit aimed to analyse the use of neuroimaging and its effect on treatment in an older adult inpatient psychiatry unit over the period of one year.

**Background:**

Brain imaging can be used as a diagnostic tool in psychiatry. According to NICE guidelines, structural imaging, such as magnetic resonance imaging (MRI) or computed topography (CT), can be used in the workup for dementia diagnosis in order to exclude non-dementia pathology and identify dementia subtype. This is important in the geriatric population where evidence of small vessel disease has an impact on treatment options and management of polypharmacy.

**Method:**

A list of patients from the past year (January to December 2019) was accessed. Patient records were then analysed to see if neuroimaging had been accessed during their admission at The Meadows Hospital, Surrey and Borders Partnership. This included imaging from prior to admission. Analysis was divided into type of imaging, comments and impact on diagnosis.

**Result:**

Overall numbers for the audit were small. A total of 74 patients were admitted onto the unit, of which 3 were readmissions. There was missing information for 8 patients, giving a total of 63 patients. CT scans were accessed for 35 patients (56% of total); 3 of these were done during the admission. MRI scans were completed for 21 patients (33%), with one requested during admission. A total of 9 patients (14%) had both CT and MRI scans. Neuroimaging results led to a change in diagnosis for 6 patients (10%). In all cases this reflected the finding of small vessel disease and a change of diagnosis to either vascular dementia or mixed dementia.

Diagnoses were also analysed. The Meadows Hospital has 2 dementia wards (male and female) and 1 functional ward (for females). A total of 36 patients (57%) were diagnosed with dementia, of which the biggest groups were: Alzheimer's dementia (13 patients, 36%) and Vascular dementia (11 patients, 31%).

**Conclusion:**

The majority of the patients were admitted with established diagnoses and so only a small number had changes made following review of imaging. Good imaging results and reports help to differentiate types of dementia. Although neuroimaging is not gold standard in diagnosing dementia syndromes, it is now an important aspect in the diagnostic pathway. Getting the diagnosis correct will help with treating individuals appropriately and avoid unnecessary prescribing.

